# Comparing Genomic and Pedigree Inbreeding Coefficients in the Slovenian Lipizzan Horse as a Case Study for Small Closed Populations

**DOI:** 10.3390/ani15192774

**Published:** 2025-09-23

**Authors:** Barbara Luštrek, Martin Šimon, Klemen Turk, Sanja Bogičević, Klemen Potočnik

**Affiliations:** 1 Department of Animal Science, Biotechnical Faculty, University of Ljubljana, Jamnikarjeva 101, 1000 Ljubljana, Slovenia; martin.simon@bf.uni-lj.si (M.Š.); sanja.bogicevic@bf.uni-lj.si (S.B.); klemen.potocnik@bf.uni-lj.si (K.P.); 2 Department for the Selective Breeding of Equidae, Clinic for Breeding and Health Care of Horses, Veterinary Faculty, University of Ljubljana, Gerbičeva 60, 1000 Ljubljana, Slovenia; klemen.turk@vf.uni-lj.si

**Keywords:** Lipizzan horse, heritage breed, conservation genetics, inbreeding, genomic inbreeding, pedigree, small population

## Abstract

In small, closed populations such as the Lipizzan horse, maintaining genetic diversity while limiting inbreeding is a key challenge in conservation breeding. The Lipizzan is an indigenous Slovenian breed with a small population and restricted gene flow from other subpopulations. Inbreeding is traditionally monitored with pedigree-based coefficients, but these often underestimate realised autozygosity, particularly when pedigree depth is limited. This study compared pedigree-based inbreeding (F_PED) with four genomic estimators (F_HOM, F_ROH, F_HBD, F_GRM) in 329 Slovenian Lipizzan horses genotyped with a 70K SNP array. Data were processed in PLINK and R. Segment-based estimators (F_ROH, F_HBD) revealed higher inbreeding than F_PED and partitioned autozygosity into recent and distant components. F_ROH identified long homozygous segments reflecting recent inbreeding, whereas HBD classification showed that most autozygosity came from distant ancestors. Correlations between pedigree- and genomic-based coefficients were moderate (ρ = −0.18–0.56), while genomic estimators showed strong agreement. These results demonstrate that genomic measures complement pedigree-based metrics by providing a fuller picture of inbreeding and its temporal origin. Incorporating genomic estimators into routine monitoring can improve mate selection, reduce inbreeding depression, and support sustainable management of genetic diversity in the Lipizzan horse, while offering a case study for other small populations with conservation goals.

## Simple Summary

The Lipizzan horse is an indigenous Slovenian breed maintained as a small, closed population with limited gene flow from other subpopulations. In such populations, safeguarding genetic diversity while controlling inbreeding is a key challenge, as excessive inbreeding may compromise fertility, health, and long-term sustainability. Traditionally, inbreeding is monitored using pedigree records, but pedigree-based estimates often underestimate the true level of relatedness. In this study, we compared pedigree-based inbreeding coefficients with genomic measures derived from SNP array data in 329 Slovenian Lipizzans. Genomic analyses can identify regions of the genome that are identical by descent (autozygosity) and distinguish whether they originate from recent or distant ancestors. Segment-based genomic methods, especially those relying on runs of homozygosity and homozygosity-by-descent, revealed higher inbreeding than pedigree estimates and indicated that most autozygosity originated from distant ancestors, with little evidence of recent close matings. Overall, our findings emphasise the value of combining pedigree and genomic information for monitoring genetic diversity. They show how genomic tools complement pedigree records, strengthen the sustainable management of the Lipizzan horse, and serve as a case study for other small, conservation-oriented populations.

## 1. Introduction

Maintaining genetic diversity while controlling inbreeding is a primary challenge in the management of small, closed horse populations (e.g., [1,2]). The Lipizzan horse, one of Europe’s oldest cultural breeds, has a complex demographic history shaped by strong founder effects, historical bottlenecks, and long-term closed studbook practices [3]. In heritage breeds, excessive inbreeding can compromise fertility, increase the risk of inherited disorders, and reduce overall viability, thereby threatening the conservation value and functional integrity of the population [4,5].

Inbreeding in such populations has traditionally been monitored using pedigree-based coefficients, which estimate the probability of identity-by-descent from known ancestors [6]. However, pedigree-based estimates can underestimate realised autozygosity, particularly when pedigrees are incomplete or when historical inbreeding extends beyond the depth of available record [7,8]. The advent of SNP genotyping has enabled more accurate quantification of realised inbreeding [9], offering approaches that can detect both recent and ancient events. These include segment-based methods such as runs of homozygosity (ROH) and hidden Markov model-based classification of homozygosity-by-descent (HBD), as well as SNP-by-SNP measures based on observed homozygosity or genomic relationship matrices [10,11,12,13]. These methods differ in their assumptions and resolution [14], and their combined use can provide a more complete picture of genetic diversity and population history, with direct applications for conservation breeding.

Each of these approaches provides complementary insights important for conserving small, closed populations. Pedigree-based coefficients (F_PED) offer a practical tool for long-term monitoring using routinely collected records, although their precision depends on both the depth and reliability of the pedigree [15]. Although the Lipizzan horse pedigree is notably deep [16], practical monitoring in breeding programmes often relies on a limited number of generations [17,18,19]. Genomic homozygosity estimates (F_HOM) offer a direct, though coarse, measure of realised inbreeding across the genome [11]. Runs of homozygosity (F_ROH) provide information on both the level and distribution of autozygosity, thereby distinguishing recent from ancient inbreeding events [20]. The genomic relationship matrix (F_GRM) is particularly useful in breeding management, as it enables integration of genomic inbreeding with relationship measures used in mate allocation [21]. Finally, homozygosity-by-descent estimates (F_HBD) refine this by assigning autozygous segments to different age classes, offering temporal resolution that can highlight historical demographic processes [22]. Together, these methods provide a comprehensive framework for monitoring genetic diversity, detecting risks of inbreeding depression, and informing sustainable mating strategies.

Although several multinational studies have examined genomic inbreeding in Lipizzans [16,23,24], the Slovenian population has not yet been evaluated in detail. Due to the historical autonomy of national breeding programmes and limited gene flow between stud farms, subpopulation-specific assessments are essential for identifying unique patterns of inbreeding and for tailoring conservation strategies to local breeding practices [25,26]. The Slovenian population is particularly relevant, as the Lipizzan horse is an indigenous breed originating from Slovenia and is managed within a closed structure comprising separate breeding programmes for the national stud farm and the private sector, both based on shared fundamental principles [3]. Given these characteristics, effective monitoring would ideally integrate regularly updated pedigree and genotypic information, quality control procedures, and calculation of both pedigree-based (F_PED) and genomic (F_ROH, F_HBD) indicators, with annual interpretation and application of results to inform mating decisions and support the sustainable management of genetic diversity. Responding to an initiative from the Slovenian Lipizzaner Breeders’ Association, we conducted this research to complement pedigree-based monitoring with genomic information for improved population management.

This study provides the first detailed comparison of pedigree-based and genomic estimates of inbreeding in the Slovenian Lipizzan horse, integrating pedigree and genomic data to assess both recent and historical autozygosity. By identifying differences in sensitivity and temporal resolution among methods, this approach delivers population-specific insights that can improve monitoring and guide evidence-based conservation breeding strategies. Such strategies are critical for sustaining genetic diversity, preventing inbreeding depression, and safeguarding the long-term genetic health and cultural heritage of the Lipizzan horse.

## 2. Materials and Methods

### 2.1. Study Population and Pedigree Data

The study was conducted on 329 genotyped Lipizzan horses from the Slovenian population, comprising 115 males and 214 females, originating from private breeders represented by the Slovenian Lipizzaner Breeders’ Association. These animals represent a subset of the Slovenian Lipizzan population, which in 2024 consisted of approximately 392 horses at the national stud farm Lipica and 756 horses in the private sector [27]. The genotyped sample was not sex-balanced, but the sex ratio and distribution of birth years reflect the availability of DNA samples.

Stallions represented six classical lines (Conversano, Favory, Maestoso, Neapolitano, Pluto, and Siglavy) and one non-classical line (Tulipan). Mares belonged to sixteen traditional mare families (Africa, Almerina, Argentina, Defloata, Djebrin, Englanderia, Europa, Gidrane, Mercurio, Munja, Presciana, Rebecca, Sardinia, Spadiglia, Stornella, and Theodorosta) and three non-classical families (Margit, Traviata, and Wanda-Mima). Horses were born between 1997 and 2024, with most born in 2017 or later.

Pedigree records were obtained from the national register of equidae (Ministry of Agriculture, Forestry and Food, Ljubljana, Slovenia), collected by two breeding organisations—Studfarm Lipica and the Slovenian Lipizzaner Breeders’ Association, and checked for data inconsistencies. Quality control included detection and correction of duplicated animal IDs, invalid sex codes, animals listed as their parent, identical sire and dam, incorrect parental sex, missing parental information, biologically implausible parental ages, and unrealistic or missing birth dates.

Pedigrees were traced over a maximum of five ancestral generations using a recursive procedure. The five-generation limit aligns with established livestock selection practices in livestock populations [28], balancing the need to avoid artificially inflated coefficients from common ancestors at greater depths with the requirement to ensure comparability of F_PED values across individuals. Incomplete parental information was tolerated to maximise sample size; however, for F_PED calculation, only animals with at least five complete ancestral generations were retained (*n* = 3487). The complete pedigree file contained 4803 animals. Generation depth for each individual was defined as the length of the longest ancestral path in the pedigree.

Pedigree analysis was performed in R version 4.4.3 [29] using the R package AGHmatrix version 2.1.4 [30]. The additive genetic relationship matrix (A) was constructed from the filtered five-generation pedigree assuming diploid inheritance, and individual F_PED values were calculated as the diagonal elements of the A matrix minus one $(F\_\mathrm{PED}\;={\;A}_{\mathrm{ii}}\;-\;1$). These coefficients were used for downstream analysis.

Pedigree-based coefficients (F_PED) were therefore computed for all animals with complete five-generation pedigrees (*n* = 3487). Still, all downstream comparisons with genomic inbreeding measures were strictly restricted to the subset of 329 genotyped horses.

This study did not involve any experimental procedures on animals. Only existing pedigree and genomic data were used.

### 2.2. Genotyping and Quality Control

Genomic DNA was extracted from hair root samples and genotyped by Neogen using the commercial GeneSeek^®^ Genomic Profiler™ Equine 70K SNP array (Illumina Equine SNP50 platform), comprising ~70,000 genome-wide markers mapped to the EquCab3.0 reference genome [31].

Quality control was performed in PLINK v1.9 [32]. SNPs with low call rate (<90%) and Hardy–Weinberg equilibrium *p*-value < 1 × 10^−6^ were excluded, as were individuals with >10% missing genotypes. Only autosomal SNPs were retained. Identity-by-state analysis (--genome command line function) identified potential duplicates or close relatives, and heterozygosity (--het command line function) was used to detect outliers. Remaining SNPs were filtered at minor allele frequency (MAF) ≥ 0.01. The final dataset comprised 56,235 autosomal SNPs and 329 genotyped animals.

### 2.3. Estimation of Genomic Inbreeding Coefficients

Four genomic inbreeding coefficients were estimated: SNP-by-SNP estimate based on observed vs. expected homozygosity (F_HOM), proportion of genome in runs of homozygosity (F_ROH), proportion of genome in homozygosity-by-descent segments inferred by hidden Markov models (F_HBD), and self-relatedness from the genomic relationship matrix (F_GRM). All genomic estimates were calculated using autosomal SNPs only.

#### 2.3.1. F_HOM

F_HOM were calculated in PLINK v1.9 using the --het command line function, following [9]. Only autosomal SNPs retained after quality control (Section 2.2) were used. For each individual i, the coefficient was calculated as:

$${F\_\mathrm{HOM}}_{i}\;=\;O\left(\mathrm{HOM}\right)\;-\;E(\mathrm{HOM})/m\;-E(\mathrm{HOM})$$

where $O\left(\mathrm{HOM}\right)$ is the observed number of homozygous genotypes, m is the total number of genotyped loci, and $E\left(\mathrm{HOM}\right)$ is the expected number of homozygotes under Hardy–Weinberg equilibrium, calculated as:

$$E\left(\mathrm{HOM}\right)\;=\sum_{j=1}^{m}\left[p_{j}^{2}+{(1-p_{j})}^{2}\right]$$

with $p_{j}$ being the frequency of the reference allele at locus j.

#### 2.3.2. F_ROH

Runs of homozygosity (ROH) were detected in PLINK v1.9 using the --homozyg command line function, following the criteria from [33] and equine-specific studies [23,34,35,36]. ROH were defined as continuous homozygous segments of at least 1 Mb in length and spanning a minimum of 15 consecutive SNPs. The maximum gap allowed between two SNPs within a segment was set to 1000 kb, and SNP density was restricted to one SNP per 200 kb. Sliding windows of 50 SNPs were applied. Each window could contain at most one heterozygous and up to five missing genotypes. A 5% threshold was used to define a homozygous run.

ROH segments were grouped into five length classes: 1–<2 Mb, 2–<4 Mb, 4–<8 Mb, 8–<16 Mb, and ≥16 Mb (e.g., [34]), reflecting different inbreeding ages. Segments ≥16 Mb represent very recent inbreeding, those 8–<16 Mb represent recent, 4–<8 Mb intermediate, and both 2–<4 Mb and 1–<2 Mb represent ancient inbreeding. ROH-based inbreeding coefficients (F_ROH) were calculated for each individual as the sum of the total ROH length divided by the estimated autosomal genome length of 2410 Mb, corresponding to the assembled genome in EquCab3.0 [31,37]. To enable classification, each ROH segment was assigned to one of the five categories based on its total physical length in Mb. For each individual, class-specific F_ROH values were calculated as the proportion of the autosomal genome covered by ROH segments within each length class, enabling the distinction between recent and ancient inbreeding.

#### 2.3.3. F_HBD

F_HBD were estimated using the R package RZooRoH version 0.4.1 [38]. The model was specified using the zoomodel() function with 10 HBD classes, defined by rate parameters increasing exponentially from R_k_ = 2 to 1024 (R_k_ = 2^i^, i = 1 to 10), as recommended by [29,30], and as applied in recent livestock studies (e.g., [39]). This approach allows the detection of autozygosity originating from a broad range of ancestral time depths, from very recent to ancient common ancestors.

The model was fitted using the zoorun() function, applying both Forward-Backwards and Viterbi algorithms to estimate the individual genomic inbreeding coefficient (F_HBD) and to identify precise HBD segment boundaries, respectively. No convergence issues were encountered, and the model yielded stable estimates of F_HBD across all individuals.

HBD segments were grouped into generational age classes based on their R_k_ parameters: R_2_–R_8_ represent very recent inbreeding, R_16_–R_64_ represent recent inbreeding, R_128_–R_256_ represent intermediate inbreeding, and R_≥512_ represent ancient inbreeding. Class-specific F_HBD values were then calculated for each individual as the proportion of the autosomal genome assigned to segments within each age group, enabling temporal partitioning of autozygosity.

#### 2.3.4. F_GRM

F_GRM were estimated based on the genomic relationship matrix (GRM), constructed using VanRaden method I [40]. The GRM was computed using the Gmatrix() function from the R package AGHmatrix version 2.1.4, assuming diploid inheritance and a centred genotype matrix. No additional minor allele frequency filtering was applied at this step to maintain consistency with the dataset used for other inbreeding coefficients. Individual F_GRM values were derived from the diagonal elements of the GRM as $F\_\mathrm{GRM}\;={\;G}_{\mathrm{ii}}-1$, where $G_{\mathrm{ii}}$ denotes the genomic self-relatedness of individual i, reflecting the deviation from the average allele frequency in the population.

### 2.4. Statistical Analysis

Descriptive statistics were computed using the describe() function from the psych package version 2.5.6 [41]. The normality of each distribution was tested using the Shapiro–Wilk test (shapiro.test()), and pairwise associations among inbreeding coefficients were assessed using Spearman’s rank correlation (cor.test() with method = “spearman”).

Simple linear regression models (lm()) were fitted with F_PED as the dependent variable and each genomic coefficient (F_HOM, F_ROH, F_HBD, and F_GRM) as an independent predictor. The results were summarised, and model diagnostics, including residual histograms and residuals vs. fitted plots, were visually examined using base R graphics to verify the assumptions of homoscedasticity and linearity. All models were interpreted based on adjusted R^2^ and *p*-values. This approach is commonly used to evaluate the concordance between pedigree-based and genomic inbreeding coefficients in livestock populations (e.g., [39,42]).

The contribution of recent vs. ancient inbreeding, ROH and HBD segments were grouped according to their predefined length or rate parameter classes, respectively (see Section 2.3.2 and Section 2.3.3). For each individual, the proportional contribution of each class to the total F_ROH or F_HBD was calculated and averaged across all genotyped animals. The distributions of segment lengths within each class were compared descriptively and visualised using grouped bar plots (geom_col(), ggplot2).

All analyses were performed in R version 4.4.3, using the dplyr version 1.1.4 [43], readr version 2.1.5 [44], tidyr version 1.3.1 [45], purrr version 1.1.0 [46], and ggplot2 version 3.5.2 [47] packages, with *p* < 0.05 considered significant.

## 3. Results

**Figure 1:**
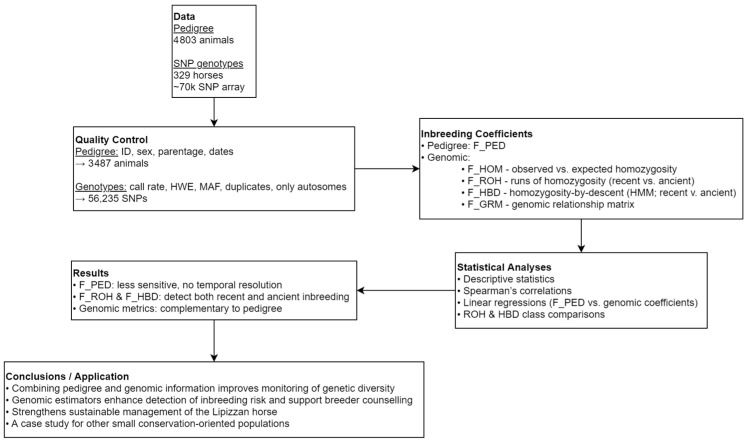
Workflow for pedigree- and genome-based inbreeding estimation in the Slovenian Lipizzan horse population.

To evaluate pedigree- and genome-based inbreeding in the Slovenian Lipizzan horse population, we combined pedigree records with genotypic data and applied quality control to ensure consistency. Four genomic inbreeding estimators (F_HOM, F_ROH, F_HBD, F_GRM) were derived, each capturing different aspects of autozygosity and enabling temporal partitioning of inbreeding. Descriptive statistics, correlations, and regression models were used to compare pedigree and genomic measures. Overall, F_PED was less sensitive and lacked temporal resolution, while F_ROH and F_HBD revealed recent versus ancient inbreeding, underscoring their complementary value for monitoring genetic diversity. The analytical workflow and main findings are illustrated in Figure 1.

### 3.1. Descriptive Statistics

Descriptive statistics for the five inbreeding coefficients are presented in Table 1. The pedigree-based coefficient (F_PED) had a mean of 0.037, with values up to 0.151 across five generations. Genomic estimates detected higher levels of autozygosity: F_HBD had the highest mean (0.177), followed by F_ROH (0.122). In contrast, F_HOM (mean = −0.022) and F_GRM (mean = −0.009) were lower on average and displayed the widest ranges, with minimum values below −0.25. This highlights their sensitivity to allele frequency variation compared with segment-based estimators.

**Figure 2:**
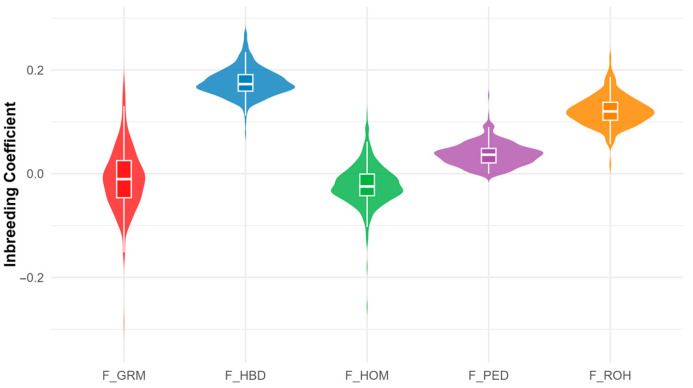
Comparison of the distribution of inbreeding coefficients estimated by different methods (F_PED: Pedigree-based inbreeding coefficient; F_ROH: Runs of homozygosity inbreeding coefficient; F_HBD: Homozygous-by-descent inbreeding coefficient; F_HOM: Homozygosity-based inbreeding coefficient; F_GRM: Genomic relationship matrix inbreeding coefficient).

Figure 2 compares the distributions of the five inbreeding coefficients. F_PED and F_ROH show relatively narrow, symmetric distributions centred on low to moderate positive values, with F_ROH shifted slightly higher. F_HBD is skewed towards higher values, indicating more inferred autozygosity. F_HOM and F_GRM have the broadest ranges, extending from substantially negative to moderately positive values, with medians slightly below zero.

**Figure 3:**
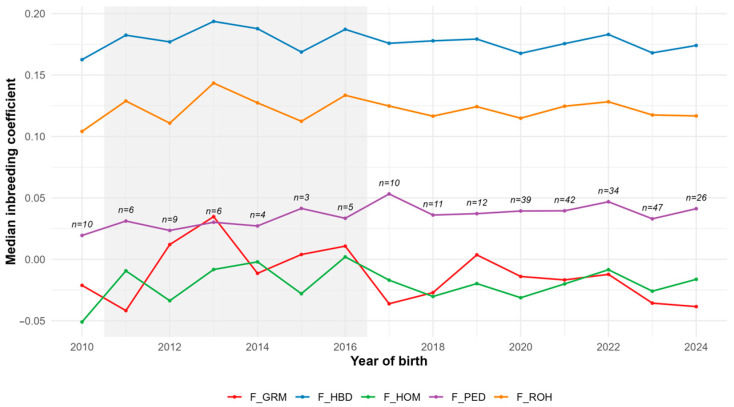
Trend of median inbreeding coefficients by year of birth (F_PED: Pedigree-based inbreeding coefficient; F_ROH: Runs of homozygosity inbreeding coefficient; F_HBD: Homozygous-by-descent inbreeding coefficient; F_HOM: Homozygosity-based inbreeding coefficient; F_GRM: Genomic relationship matrix inbreeding coefficient).

Median inbreeding coefficients by birth year (2010–2024) are shown in Figure 3. F_HBD remained consistently highest (~0.17–0.19) with slight temporal fluctuation, while F_ROH was lower but similarly stable. F_PED showed modest inter-cohort variation without a clear temporal trend. F_HOM and F_GRM displayed greater variability, including negative medians in some years, which partly reflects small sample sizes (*n* < 10 for some cohorts).

### 3.2. Normality Test and Pairwise Associations

All coefficients deviated significantly from normality (Shapiro–Wilk tests, *p* < 0.001), justifying the use of non-parametric correlations. Pairwise associations revealed that F_PED correlated most strongly with F_ROH (ρ = 0.562), while correlations with F_HOM and F_HBD were moderate (ρ = 0.496 and 0.469, respectively). A weak but significant negative correlation was observed with F_GRM (ρ = −0.182). Among genomic estimators, correlations were very high between F_ROH, F_HOM, and F_HBD (all ρ > 0.9), whereas associations with F_GRM were consistently weaker (Table 2). All correlations were statistically significant (*p* < 0.01).

### 3.3. Linear Regression Analysis

**Figure 4:**
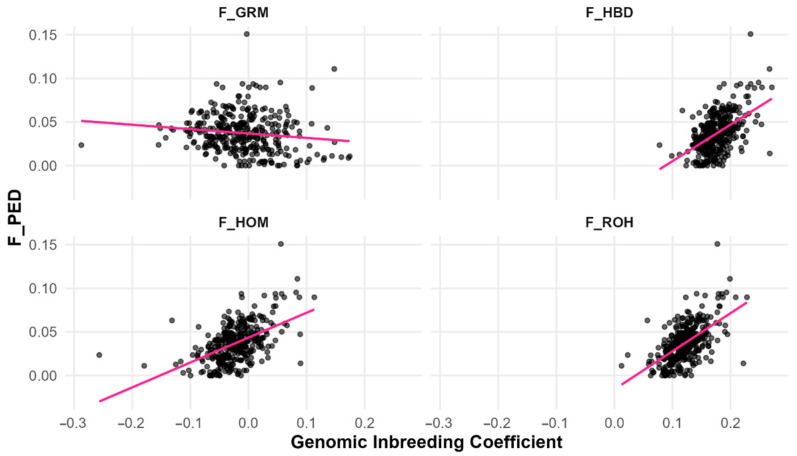
Linear regression of the pedigree-based inbreeding coefficient on four genomic inbreeding coefficients, corresponding to regression models in Table 3. The dots represent individual animals (observed values), and the line represents the fitted regression line (F_PED: Pedigree-based inbreeding coefficient; F_ROH: Runs of homozygosity inbreeding coefficient; F_HBD: Homozygous-by-descent inbreeding coefficient; F_HOM: Homozygosity-based inbreeding coefficient; F_GRM: Genomic relationship matrix inbreeding coefficient).

**Figure A1:**
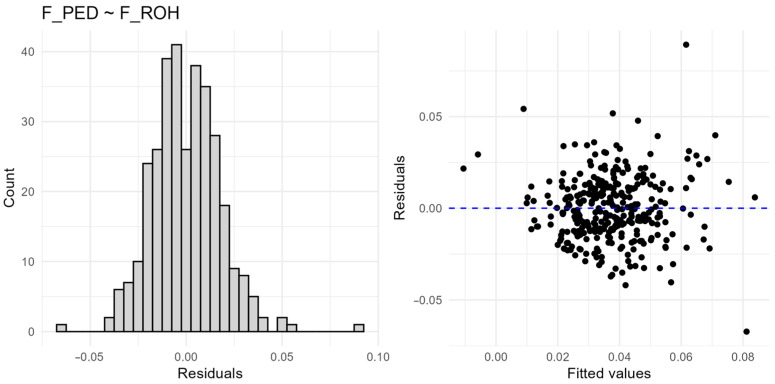
Model diagnostics for the regression of pedigree-based inbreeding coefficient (F_PED) on runs of homozygosity inbreeding coefficient (F_ROH). (**Left**) histogram of model residuals showing approximate normal distribution. (**Right**) residuals vs. fitted values; the dots represent individual observations and the dashed line indicates the fitted trend, showing no strong evidence of heteroscedasticity.

**Figure A2:**
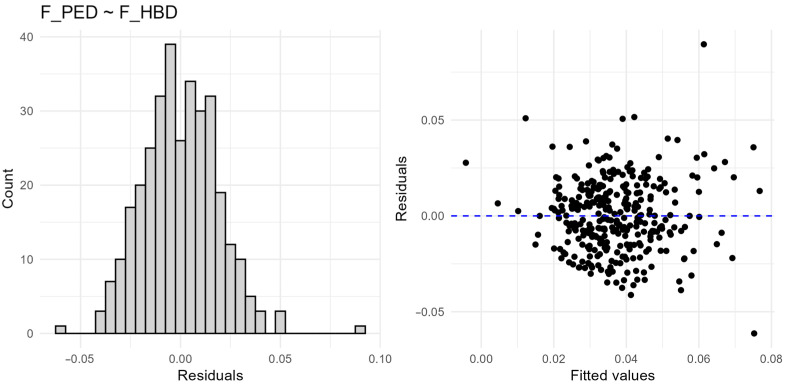
Model diagnostics for the regression of pedigree-based inbreeding coefficient (F_PED) on homozygosity-by-descent inbreeding coefficient (F_HBD). (**Left**) histogram of model residuals showing approximate normal distribution. (**Right**) residuals vs. fitted values; the dots represent individual observations and the dashed line indicates the fitted trend, showing no strong evidence of heteroscedasticity.

Linear regressions confirmed that segment-based metrics (F_ROH, F_HBD) and F_HOM better predicted F_PED than allele frequency-based F_GRM (Table 3; Figure 4). F_ROH explained the highest proportion of variance in F_PED (R^2^ = 0.337), followed by F_HBD (R^2^ = 0.265), and F_HOM (R^2^ = 0.262). In contrast, F_GRM had negligible predictive value (R^2^ = 0.016). These results demonstrate that measures based on genomic segments provide a closer reflection of pedigree-based inbreeding than allele frequency-based estimators. Model diagnostics revealed no violations of linear model assumptions (model diagnostics are provided in Appendix A (Figure A1 and Figure A2).

### 3.4. ROH and HBD Length Category Contributions

A total of 15,973 ROH segments were detected, corresponding to an average of ~49 per individual. Partitioning of autozygosity by segment length and HBD class showed that most inbreeding originated from intermediate and ancient ancestors. Short ROH segments (1–<4 Mb) were the most numerous, whereas very long segments (≥16 Mb) were rare, indicating limited recent inbreeding. HBD classification showed a similar distribution, with the majority of segments assigned to intermediate classes (R_16_–R_256_) and only a few in the very recent categories (R_2_–R_4_). Mean segment length declined with increasing ancestral age, i.e., with segments tracing back to progressively more distant common ancestors, from >50 Mb in the very recent HBD classes to <1 Mb in the most ancient class (R_512_). Overall, both ROH- and HBD-based partitioning pointed to the predominance of autozygosity originating from distant rather than recent common ancestors (Table 4).

**Figure 5:**
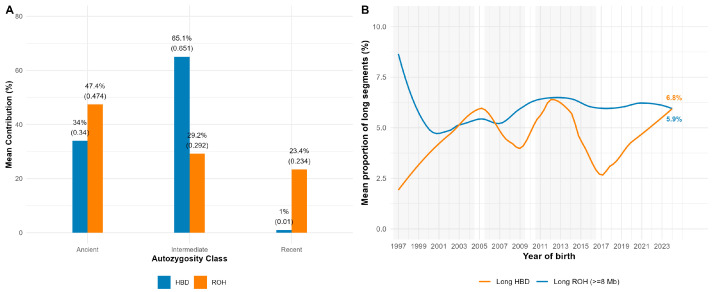
Relative contributions of ancient, intermediate, and recent autozygosity and their temporal dynamics. (**A**) Partitioning of autozygosity by age class; (**B**) temporal trends in long segments.

Figure 5 shows the partitioning of autozygosity by age class (A) and temporal trends (B) in long segments. ROH-based estimates attributed 47.4% of total F_ROH to ancient segments (1–<4 Mb), 29.2% to intermediate segments (4–<8 Mb), and 23.4% to recent segments (≥8 Mb). HBD-based estimates showed a consistent pattern, with 65.1% of F_HBD assigned to intermediate classes (R_8_–R_32_), 34% to ancient classes (R_64_–R_1024_), and only ~1% to very recent classes (R_2_–R_4_). Thus, both approaches indicate that the majority of inbreeding originated from earlier generations.

The proportion of long ROH segments (≥8 Mb) remained relatively stable over the past two decades (5.9% for ROH and 6.8% for HBD). Although HBD trends were more variable in years with smaller sample sizes, neither method showed evidence of a persistent upward trajectory in recent inbreeding.

## 4. Discussion

In the Slovenian Lipizzan population, F_PED (mean = 0.037 over five generations) clearly underestimated overall inbreeding, particularly that originating from more distant common ancestors [4,9]. This is consistent with previous studies showing that limited pedigree depth and incomplete ancestral records lead to downward-biassed estimates, especially in small, closed populations, as also observed in Polish and Italian draft horses [35,48]. Because pedigree-based coefficients are typically restricted to a few generations in routine selection practices (e.g., [19]), they mainly capture recent shared ancestry and cannot account for older autozygosity. In contrast, genomic approaches such as ROH and HBD directly identify autozygous segments across the genome, thereby detecting both recent and ancient inbreeding [20,22]. The predominance of short ROH and HBD segments in our data illustrates this older component, which is invisible to shallow pedigrees but contributes substantially to realised autozygosity.

Segment-based measures also captured broader signals of realised inbreeding. In our study, mean F_HBD (0.177) exceeded F_ROH (0.122), both robust to pedigree incompleteness and consistent with earlier simulation-based findings [14] and applications in wild populations [25]. Documented discrepancies between pedigree records and Y-chromosomal haplotypes in Lipizzan classical sire lines such as Maestoso, Siglavy and Favory underscore the importance of genetic validation of historical genealogies [49]. The commercial SNP array used in this study includes a few Y-linked markers from the EMSY region, but coverage is sparse and uninformative; in line with standard practice, only autosomal SNPs were used for genomic inbreeding estimation. Similar pedigree-genomic mismatches, due to founder substitutions or misassigned paternity, have been reported in other horse and livestock populations [50,51,52,53]. In Anglo-Arabians, for example, genomic inbreeding (F_ROH = 21.1%) far exceeded pedigree-based estimates (F_PED = 0.7%) due to performance selection and shallow pedigrees [54].

SNP-by-SNP estimates (F_HOM = −0.022 and F_GRM = −0.009 on average) showed wide ranges, including negatives, reflecting sensitivity to allele frequency distributions and deviations from the Hardy–Weinberg equilibrium in small structured populations. Consequently, they correlated only weakly with pedigree-based measures. The negative correlations of F_GRM with F_PED reflect methodological differences rather than pedigree errors: F_PED estimates identity-by-descent within five generations, while F_GRM measures self-relatedness relative to population allele frequencies. Similar discrepancies have been reported in cattle and horse populations [34,36,40,55]. In contrast, segment-based measures such as F_ROH are less affected by allele frequency biases [56,57] and therefore provide greater robustness and accuracy, particularly in small or structured populations such as heritage breeds [6].

A meta-analysis [58] found that SNP-based coefficients, particularly F_HOM and F_GRM, were more strongly linked to inbreeding depression than pedigree-based estimates, reflecting their sensitivity to recent consanguinity but with greater variability. In Pura Raza Española horses, only genomic measures (F_ROH, F_HBD), but not F_PED, were associated with fertility traits, consistent with results observed in Japanese Black cattle and Austrian Lipizzans [23,39,59].

Among our correlations, F_ROH showed the strongest association with F_PED (ρ = 0.562), consistent with its ability to capture long homozygous segments indicative of recent common ancestry [60]. In contrast, F_GRM correlated weakly and negatively with F_PED (ρ = −0.182), reflecting allele frequency biases in small, structured populations [61,62], a pattern similarly reported in cattle and local horse breeds [34,36,55]. Genomic estimators themselves were strongly correlated (ρ = 0.927–0.941), with F_HBD and F_HOM showing near-perfect agreement (ρ = 0.932) and both closely matching F_ROH, in line with their shared focus on realised homozygosity and segment-level autozygosity [9,20].

Regression analyses confirmed that segment-based measures explain more variation in F_PED than frequency-based ones (F_ROH R^2^ = 0.337; F_HBD R^2^ = 0.265; F_HOM R^2^ = 0.262 vs. F_GRM R^2^ = 0.016), highlighting the greater suitability of segment-based approaches for capturing realised inbreeding in small, closed populations [48,63]. Yet these modest R^2^ values show that substantial variation remains unexplained. Likely contributors include the shallow pedigree depth (capturing only recent ancestry), stochastic variation from recombination and Mendelian sampling, potential pedigree errors, methodological differences between recorded-ancestry expectations and genomic IBD, and technical limits of medium-density SNP arrays, which miss very short or very long segments. Allele frequency structure and LD patterns in small closed populations further add to these discrepancies.

Partitioning F_ROH by segment length indicated that short ROH (1–4 Mb), reflecting ancient inbreeding from long-term small effective population size and isolation [64,65], accounted for nearly half of the total F_ROH. In contrast, long ROH (≥8 Mb), indicative of recent inbreeding, contributed only 23% [64,66]. This aligns with the expected exponential decline in segment length with generational distance, documented in livestock [67] and in ancient human genomes [68].

Similar patterns occur in Quarter Horses, where over half of the segments are 1–2 Mb and very few exceed 16 Mb, indicating predominantly ancient autozygosity [69]. In contrast, Thoroughbreds show rising inbreeding due to popular sire use and closed studbooks [70,71]. Comparable dynamics are seen in other closed breeds, including the Norik of Muran horse [72]. While the Lipizzan population is also closed, our results show that most autozygosity originates from distant rather than recent ancestors, with no evidence of a comparable recent increase.

The HBD profile confirmed this pattern, with >98% of F_HBD from intermediate and ancient classes (R_32_–R_1024_) and negligible recent contribution (<0.1%). Such historical bottlenecks are consistent with reports in the TRNP feral horses [73] and Belgian Blue cattle [74]. The predominance of historical inbreeding likely reflects long-term closure, reinforced by strict pedigree-based conservation and molecular verification in Italian Lipizzans [75]. Technical factors also apply: medium-density SNP arrays underestimate very short ROH and may fail to detect very long HBD [76,77]. Nevertheless, temporal analysis showed no increase in close inbreeding over the past two decades, with long ROH and long HBD averaging 5.9% and 6.8% in the youngest cohort. Differences between ROH and HBD highlight their complementarity: ROH thresholds detect long recent segments, while HBD probabilistic models better capture short ancient ones [78,79,80]. Their alignment supports using both methods to date inbreeding events [22,23,50,81].

Taken together, these findings have direct implications for conservation breeding. From a management perspective, segment-based genomic metrics provide valuable resolution for detecting bottlenecks and consanguinity overlooked by pedigrees and should be incorporated into routine Lipizzan breeding programmes [80]. In practice, animals with disproportionately high levels of long ROH or recent HBD segments can be prioritised for outcrossing. At the same time, those with lower genomic inbreeding may be used more widely without increasing autozygosity. Such indicators can complement existing pedigree-based monitoring and give breeders an additional genomic perspective when planning matings [82].

This study has several limitations. First, F_PED was calculated over five generations, following established livestock practice where shallow pedigrees used in routine selection avoid inflated inbreeding values but miss older autozygosity. Second, the SNP chip contained only a few Y-linked SNP markers; thus, only autosomal SNPs were used, precluding validation of sire line integrity. Third, medium-density SNP array (~70 K) constrains ROH detection, underestimating very short or very long segments [83,84]. Fourth, pedigree errors such as misassigned parentage cannot be excluded and may contribute to discrepancies between pedigree and genomic coefficients. Finally, the 329 genotyped horses represented all available SNP data rather than a systematically balanced sample. It should also be noted that although F_PED was computed for all horses with complete five-generation pedigrees, all pedigree-genomic comparisons were restricted to the 329 genotyped animals, avoiding systematic bias.

Despite these limitations, the integration of pedigree and genomic information provides valuable insights into inbreeding dynamics and a practical framework for conservation management of the Lipizzan horse. Beyond genetics, preserving the Lipizzan horse also requires safeguarding its cultural and historical heritage, as seen in Hungary, where unbalanced representation of mare families threatens both genetic diversity and breed identity [85]. Maintaining genomic diversity is therefore vital not only for biological resilience but also for cultural preservation. Integrating genomic data from the Slovenian Lipizzaner Breeders’ Association with those from Studfarm Lipica would further strengthen national monitoring, while international collaboration could enhance global conservation management, given the small census size of the breed.

Future research should evaluate whether autozygous segments overlap with known QTL or genomic regions under selection, particularly those associated with fertility, health, or morphology. The use of high-density SNP arrays or whole-genome sequencing (WGS) will further improve precision and enable the identification of functionally relevant regions [70,84,86,87], while parameters such as effective population size derived from LD could provide additional insight into long-term management.

Beyond the Lipizzan horse, the analytical framework developed is broadly applicable. It may serve as a practical tool for managing genetic diversity in other small populations, thereby contributing to the sustainable conservation of livestock and wildlife genetic resources.

Overall, our findings emphasise the value of combining pedigree and genomic information for monitoring genetic diversity and demonstrate how genomic tools can strengthen the sustainable management of the Lipizzan horse and serve as a case study for other small, conservation-oriented populations.

## 5. Conclusions

This study provides the first detailed comparison of pedigree-based (F_PED) and genomic inbreeding coefficients (F_HOM, F_ROH, F_HBD, F_GRM) in the Slovenian Lipizzan horse population, focusing on animals from the private breeding sector. Pedigree and genomic analyses revealed complementary insights, with segment-based metrics (F_ROH, F_HBD) proving more effective in detecting both recent and historical autozygosity. These approaches offer temporal resolution and individual-level accuracy that cannot be achieved using pedigree data alone, making them highly suitable for small, closed populations.

We recommend incorporating genomic estimators into national breeding programmes to complement pedigree-based monitoring. Their routine use can reduce the risk of inbreeding depression through informed mate selection and avoidance of unintended close matings. Adoption of these tools will strengthen sustainable management and secure the long-term genetic health of the Lipizzan horse, preserving not only its biological resilience but also its cultural and historical heritage.

## Figures and Tables

**Table 1:** Summary statistics for pedigree- and genomics-based inbreeding coefficients in Slovenian Lipizzan horses.

Inbreeding Coefficient	Mean	Standard Deviation	Minimum	Maximum
F_PED	0.037	0.022	0	0.151
F_HOM	−0.022	0.040	−0.257	0.113
F_ROH	0.122	0.029	0.012	0.228
F_HBD	0.177	0.027	0.078	0.271
F_GRM	−0.009	0.059	−0.288	0.174

F_PED: Pedigree-based inbreeding coefficient; F_ROH: Runs of homozygosity inbreeding coefficient; F_HBD: Homozygous-by-descent inbreeding coefficient; F_HOM: Homozygosity-based inbreeding coefficient; F_GRM: Genomic relationship matrix inbreeding coefficient.

**Table 2:** Spearman’s rank correlation coefficients (ρ) between pedigree-based and genomic inbreeding coefficients in Slovenian Lipizzan horses (*n* = 329).

	F_HOM	F_ROH	F_HBD	F_GRM
F_PED	0.496	0.562	0.469	−0.182
F_HOM		0.927	0.932	0.173
F_ROH			0.941	0.153
F_HBD				0.353

F_PED: Pedigree-based inbreeding coefficient; F_ROH: Runs of homozygosity inbreeding coefficient; F_HBD: Homozygous-by-descent inbreeding coefficient; F_HOM: Homozygosity-based inbreeding coefficient; F_GRM: Genomic relationship matrix inbreeding coefficient.

**Table 3:** Linear regression models assessing the association between the pedigree-based inbreeding coefficient (F_PED) and four genomic inbreeding coefficients (F_HOM, F_ROH, F_HBD, F_GRM).

Model	β	R^2^	*p*-Value
F_PED~F_HOM	0.285	0.262	<0.001
F_PED~F_ROH	0.437	0.337	<0.001
F_PED~F_HBD	0.419	0.265	<0.001
F_PED~F_GRM	−0.050	0.016	0.014

F_PED: Pedigree-based inbreeding coefficient; F_ROH: Runs of homozygosity inbreeding coefficient; F_HBD: Homozygous-by-descent inbreeding coefficient; F_HOM: Homozygosity-based inbreeding coefficient; F_GRM: Genomic relationship matrix inbreeding coefficient.

**Table 4:** Proportional contributions of HBD age classes and ROH length categories to total autozygosity (F_ROH and F_HBD), grouped by inbreeding age.

Segment Type	Class	Inbreeding Age	N Segments	Min Length (Mb)	Max Length (Mb)	Mean Length (Mb)
HBD	R_2_	Very recent	3	32.56	75.59	52.65
	R_4_	Very recent	31	9.41	67.24	36.69
	R_8_	Very recent	567	1.43	57.40	19.74
	R_16_	Recent	3746	0.59	46.21	9.08
	R_32_	Recent	10,245	0.33	23.03	4.31
	R_64_	Recent	2259	0.01	9.21	2.45
	R_128_	Intermediate	1327	0.15	3.48	1.47
	R_256_	Intermediate	19,029	0.00005	2.19	0.74
	R_512_	Ancient	1925	0.000001	1.11	0.32
ROH	≥16	Very recent	296	16.04	258.26	69.61
	8–<16	Recent	327	8.66	176.93	82.99
	4–<8	Intermediate	329	9.73	152.23	83.34
	2–<4	Ancient	329	18.02	101.31	57.97
	1–<2	Ancient	319	1.24	16.28	6.61

## Data Availability

The genotypic and pedigree data used in this study are not publicly available due to data protection agreements with the breeding organisation. However, they may be made available by the corresponding author upon reasonable request and with permission from the data owner. Summary statistics and R scripts used for inbreeding coefficient estimation are available from the authors upon request.

## Abbreviations

The following abbreviations are used in this manuscript:

F_PED	Pedigree-based inbreeding coefficient (based on recorded ancestry)
ROH	Runs of homozygosity (continuous homozygous genomic segments)
F_ROH	Inbreeding coefficient estimated from the proportion of the genome covered by ROH
F_HOM	SNP-based inbreeding coefficient estimated from observed versus expected homozygosity
F_HBD	Inbreeding coefficient estimated based on homozygosity-by-descent using a hidden Markov model
F_GRM	Inbreeding coefficient derived from the diagonal of the genomic relationship matrix
GRM	Genomic relationship matrix (based on SNP allele sharing between individuals)
